# Characteration and comparative analysis of the whole chloroplast genomes of five common millet (*Panicum miliaceum*)

**DOI:** 10.1080/23802359.2020.1866452

**Published:** 2021-03-11

**Authors:** Xiaoning Cao, Sichen Liu, Zhixin Mu, Zhijun Qiao

**Affiliations:** a Center for Agricultural Genetic Resources Research, Shanxi Agricultural University, Taiyuan, China; b Key Laboratory of Crop Gene Resources and Germplasm Enhancement on Loess Plateau, Ministry of Agriculture, Taiyuan, Shanxi, China; c Shanxi Key Laboratory of Genetic Resources and Genetic Improvement of Minor Crops, Taiyuan, Shanxi, China

**Keywords:** Common millet, cultivars, microsatellites, Gramineae, complete chloroplast genomes

## Abstract

Common millet (*Panicum miliaceum*) is the most valuable and ancient domesticated important crops in the world. We compared five common millet complete chloroplast genomes. A complete map of the variability across the genomes of the five common millet was produced that included single nucleotide variants, InDels, and structural variants, as well as differences in simple sequence repeats and repeat sequences. Molecular phylogeny strongly supported division of the five walnut species into single monophyly with a 100% bootstrap value. The availability of these genomes will provide genetic information for identifying species and hybrids, taxonomy, phylogeny, and evolution in common millet.

## 1. Introduction

Common Millet (or broomcorn millet; *Panicum miliaceum*) was one of the most important and ancient domesticated crops in the world (Lu et al. 2009). They were the staple foods in the semiarid regions of East Asia (China, Japan, Russia, India, and Korea) and even in the entire Eurasian continent before the popularity of rice and wheat (Fuller 2006), and are still important foods in these regions today (Li and Wu 1996; Lu et al. 2009).

Chloroplasts play an important role in photosynthesis in green plants and participate in the biosynthesis of starch, fatty acids, and amino acids (Neuhaus and Emes 2000). The plant chloroplast genome consists of two large inverted repeats separated by a large single copy (LSC) region and a small single copy (SSC) region (Palmer 1985). The DNA sequence of the chloroplast genome can be used as a super barcode or a resource for research in phylogeography, genetic diversity, and evolution. Although many members of the Gramineae are economically important and had complete chloroplast genome data available on a public database on a website, the common millet (*P. miliaceum*) has no complete chloroplast sequence published.

In this study, we combined *de novo* and reference-guided assembly of five common millet cultivars’ whole chloroplast genomes (whole chloroplast genomes). This is the first comprehensive Cpg (chloroplast genomes) analysis of multiple common millet cultivars from different locations. Our aims were: (1) to investigate global structural patterns of whole chloroplast genomes of five common millet cultivars including genome structure, gene order, and gene content; (2) to examine variations of simple sequence repeats (SSRs) and large repeat sequence in the whole chloroplast genomes of common millet cultivars; (3) to identify divergence hotspots as regions potentially under selection pressure; and (4) to construct a chloroplast phylogeny for the five common millet cultivars using their whole cp (chloroplast) DNA sequences.

## 2. Materials and methods

### 2.1. Taxon sampling and plant material

Fresh leaves of five common millet cultivars were collected from different locations in China and United States, including a cultivar (00007664, I8) plant growing in the USA, a native variety (S5) from Hequ, Shanxi is located near the junction between the Loess Plateau and the North China Plain at an elevation of 260–270 m, a cultivar (S9) collected from Tibet, a cultivar (‘Nianfeng 5’) collected from Heilongjiang Academy of Agricultural Sciences, and a wild common millet collected from field at Hequ, Shanxi which is located near the junction between the Loess Plateau and the North China Plain at an elevation of 260–270 m (Table 1). We stored the plant samples and specimen at Shanxi Agricultural University. The numbers of the specimen were SAU20180010LY, SAU20180011LY, SAU20180012LY, SAU20180013LY, and SAU20180014LY, respectively. The leaves were dried in silica gel and stored at −4 °C. High-quality genomic DNA was extracted using a modified CTAB method (Doyle and Doyle 1987). A paired-end (PE) library with 350-bp insert size was constructed using the Illumina PE DNA library kit according to the manufacturer’s instructions and sequenced using an Illumina Hiseq2500 by Novogene (www.novogene.com, China). The DNA concentration was quantified using a NanoDrop spectrophotometer (Thermo Scientific, Carlsbad, CA, USA). The final DNA concentration, >50 ng μL^−1^, was chosen for further Illumina sequencing. We sequenced the complete chloroplast genome of wild common millet with the Illumina sequencing platform (www.novogene.com, China). We assembled the chloroplast genomes using SPAdes (Bankevich et al. 2012) and annotated them with CpGAVAS (Liu et al. 2012). We sequenced the complete chloroplast genome of other four common millet using Illumina HiSeq 2500 sequencing technology via a combination of *de novo* and reference-guided assembly Based on the complete chloroplast genome of wild common millet as a reference, we sequenced a total of five common millet using Illumina HiSeq 2500 platform with a combination of *de novo* and reference-guided assembly.

**Table 1.** Five individuals from four locations of *P. miliaceum* used in this study.

Collection site	Name	Species	Longitude (E)	Latitude (N)	Elevation (m)
Hequ, Shanxi	Wild common millet	*P. miliaceum*	111°14′	39°08′	210
Hequ, Shanxi	s5-Hongmizi	*P. miliaceum*	115°91′	40°18′	260
United States	C-I8-USA	*P. miliaceum*	127°70′	41°56′	ND
Heilongjiang	C-s4-Nianfeng	*P. miliaceum*	118°08′	40°50′	ND
Tibet	C-s9-Tibet	*P. miliaceum*	115°85′	39°97′	2267

*Note*: ‘C’ indicates cultivars of *P. miliaceum*; ND indicates data was not available; NCBI NO. indicates Genbank accession numbers on NCBI.

### 2.2. Chloroplast genome sequencing, assembly, and gap filling

Raw reads with sequences shorter than 50 bp or with more than the allowed maximum percentage of ambiguous bases (2%) were removed from the total next-generation sequencing (NGS) PE reads using the NGSQC toolkit trim tool. After trimming, high-quality PE reads were assembled using MIRA (Chevreux et al. 2004) assembler. Then, to further assemble the complete chloroplast genome, some ambiguous regions were picked out for extension with a baiting and iteration method based on MITObim . A *de novo* assembly strategy combined with a reference-based assembly allowed us to reconstruct each complete chloroplast genome. Reads were then remapped to references for each taxon to check for mis-assemblies or rearrangements using Geneious (Kearse et al. 2012) and read matching the draft reference were assembled *de novo*, also in Geneious, using suggested settings. Inverted repeat boundaries were determined and verified by remapping reads in Geneious.

### 2.3. Genome annotation and analysis

The completed genome sequences were imported into the online program Dual Organellar Genome Annotator (Wyman et al. 2004) for annotation, coupled with manual investigation of the positions of start and stop codons and boundaries between introns and exons. Putative starts, stops, and intron positions were determined by the comparison between homologous genes and other chloroplast genomes using MAFFT (Katoh and Standley 2013). Genes and open reading frames (ORF) that may not have been annotated were identified with the aid of Geneious. In addition, all tRNA genes were further verified online using tRNAscan-SE search server (Lowe and Eddy 1997). The circular *P. miliaceum* (I8) chloroplast genome map was drawn using Organellar Genome DRAW . Genome annotation was performed in Geneious, and the GC-content of protein-coding genes, tRNA genes, introns, and intergenic spacers (IGSs) was determined on the basis of their annotation. Complete chloroplast genome comparison among the five *P. miliaceum* was performed with VISTA (Frazer et al. 2004).

### 2.4. Repeat sequencing analysis

The genomic sequences were analyzed to identify potential microsatellites (simple sequence repeats or SSRs, i.e. mono-, di-, tri-, tetra-, penta-, and hexanucleotide repeats) using MISA software (http://pgrc.ipk-gatersleben.de/misa/) with thresholds of ten repeat units for mononucleotide SSRs and five repeat units for di-, tri-, tetra-, penta-, and hexanucleotide SSRs. The web-based software REPuter (Kurtz et al. 2001) was used to analyze the repeat sequences, which included forward, reverse, complement, palindromic, and tandem repeats with minimal lengths of 30 bp and edit distances of less than 3 bp. The large repeat sequences were analyzed by using the Web-based Tandem Repeats Finder (Benson 1999). We investigated if the repeated elements identified in the chloroplast of *P. miliaceum* were also present in the other four *P. miliaceum* plants by aligning their cp genomes using Geneious (Kearse et al. 2012). Tandem repeat sequences (>10 bp in length) were detected using the online program Tandem Repeats Finder (Benson 1999), with 2, 7, and 7 set for the alignment parameters match, mismatch, and indel, respectively. The minimum alignments core and maximum period size were 80 and 500, respectively.

### 2.5. Phylogenetic analysis

The five common millet complete chloroplast genome sequences from the data-complete data set were aligned with MAFFT (Katoh and Standley 2013). The analysis was carried out based on the complete cp DNA sequences. The maximum-likelihood (ML) phylogenetic tree analysis was performed using RAxML (Stamatakis 2014), and branch support was estimated with 1000 bootstrap replicates. The phylogenetic analysis was carried out using the complete chloroplast genome sequences of all five common millet plus nine other species with complete chloroplast genome sequences (*Zea mays* (KF241981); Sorghum bicolor (EF115542); *Panicum virgatum* (HQ731441); *P. virgatum* (NC_015990); *P. virgatum* (HQ822121); *Panicum sumatrense* (NC_0322378); *P. miliaceum* (NC_029732); *Oryza rufipogon* (KF562709); *Anomochloa marantoidea* (NC_014062); *Bambumsa oldhamii* (NC_012927); Table S2).

## 3. Results and discussion

### 3.1. Genome assembly and characters of common millet complete chloroplast genome sequences

**Figure 1.**
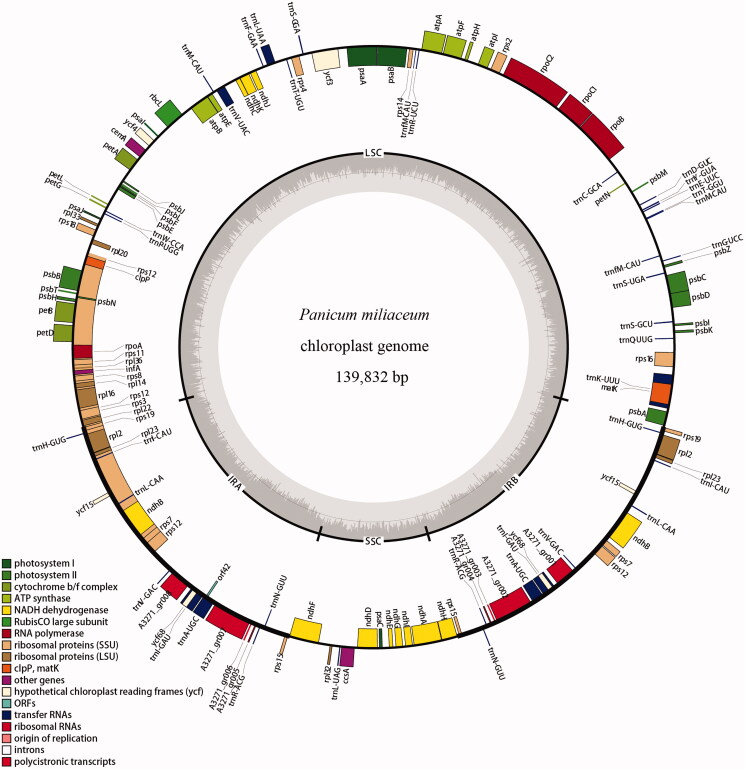
Chloroplast genome maps of common millet (*P. miliaceum*). Genes drawn outside the outer circle are transcribed clockwise, and those inside are transcribed counter-clockwise. Genes belonging to different functional groups are color coded. The dark gray in the inner circle indicates GC content of the chloroplast genomes.

The complete chloroplast genome sequence of *P. miliaceum* was 139,929 bp in length. The GC content was 38.6%. Chloroplasts are typically AT-rich, and the GC content of the *P. miliaceum* chloroplast was similar to values previously reported for most other Gramineae species (e.g. 39.0% in *Chikusichloa aquatica*; Zhang et al. 2015). The LSC and SSC contained 81,918 bp and 12,565 bp, respectively, while the IR was 22,723 bp in length. This chloroplast genome contained 132 functional genes, including 84 protein-coding genes, 30 tRNA genes, and 8 rRNA genes. There were 14 protein-coding genes, 16 tRNA, and all 8 rRNA genes duplicated in the IR regions. The LSC region contained 60 protein-coding and 23 tRNA genes, whereas the SSC region contained 10 protein-coding and one tRNA gene (Figure 1). Fourteen genes contained one or two introns, including the protein-coding genes, *rps16*, *atpF*, *ycf3* (three introns), *petB*, *petD*, *rpl16*, *rpl2*, *ndhB*, *ndhA*, and *rps12*.

### 3.2. Comparing five common millet complete chloroplast genome sequences

**Figure 2.**
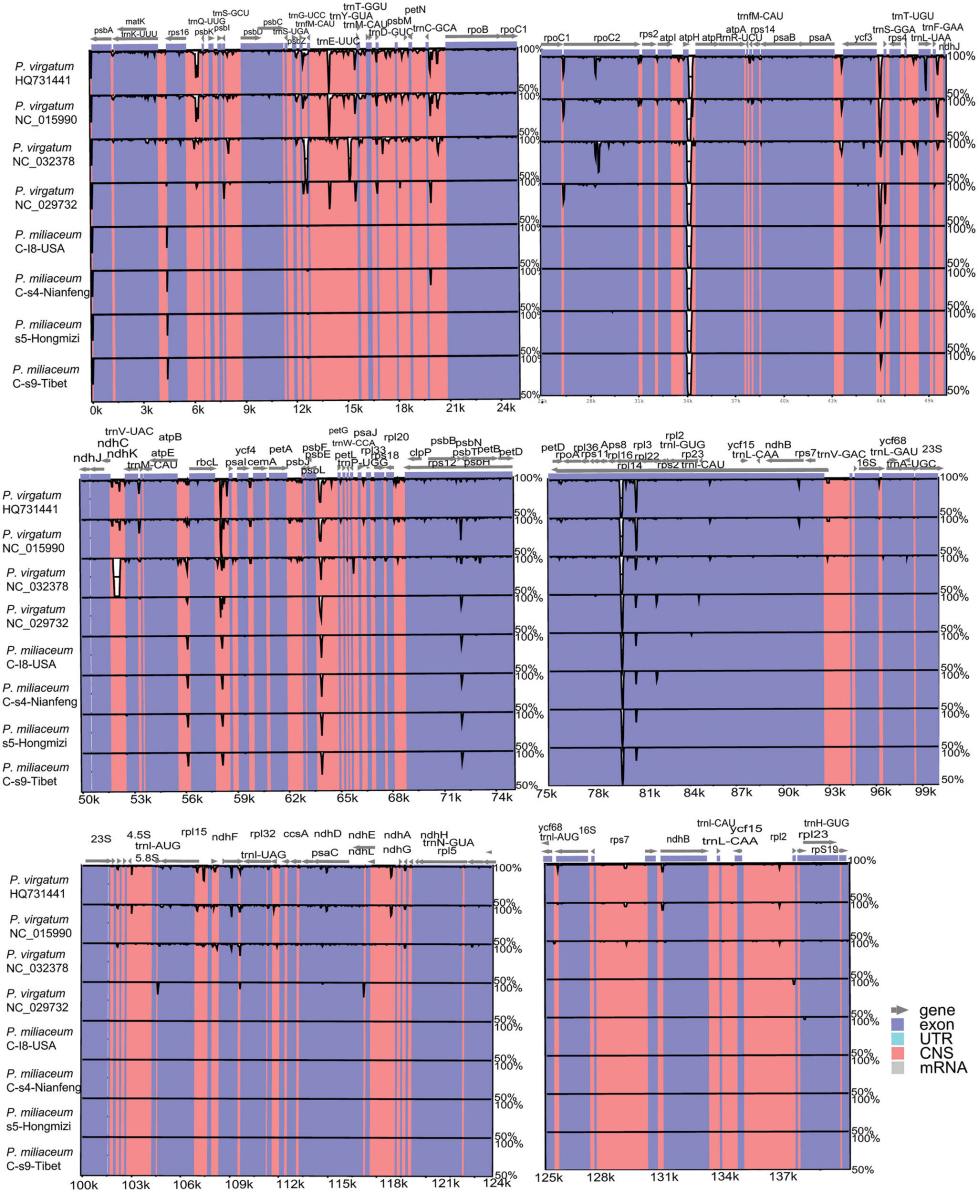
Sequence identity plot comparing the five Common millet (*P. miliaceum*) chloroplast genomes with wild as a reference by using mVISTA. Vertical scale indicates the percentage of identity ranging from 50 to 100%. Coding regions are marked in blue and non-coding regions are marked in red. Gray arrows indicate the position and direction of each gene.

We observed differences between five common millet complete chloroplast genome sequences and those of *P. virgatum* (HQ731441), *P. virgatum* (NC_015990), *P. sumatrense* (NC_032378), and *P. miliaceum* (NC_029732), two closely related to the genus *Panicum* (Figure 2). When duplicated genes in IR regions were counted only once, all five common millet complete chloroplast genome sequences harbored 80 protein-coding genes arranged in the same order (Table 2). All five common millet IR regions of the complete chloroplast genome were well conserved, including gene number and gene order, but they exhibited obvious differences at the single-copy (SC) boundary regions (Figure 2). The length of IR regions and SSC regions of nucleotide sequence were the same among the five species, especially the IR regions was 22,723 bp. (Table 1 and Figure 1). We found that the nucleotide sequence differences were mainly among the members of the genus *Panicum* (*P. miliaceum*, *P. virgatum*, HQ731441; and *P. sumatrense*, NC_032378; Figure 2). A total of 21 genes region had existed highly variation between *P. miliaceum* and other two closed relative species *P. virgatum* and *P. sumatrense* (Figure 2). We also identified five inter-space regions and four gene regions that existed highly differences of nucleotide sequence between wild *P. miliaceum* and four cultivars of *P. miliaceum* (Figure 2), such as trnfM-CAU, trnC-GCA, atpH, trnS-GGA, and trnL-UAA.

**Table 2.** Gene contents in five *P. miliaceum* chloroplast genomes.

Category of genes	Group of gene					
Self-replication	Ribosomal RNA genes	*rrn4.5*^a^	*rrna5*^a^	*rrn16*^a^	*rrn23*^a^	
	Transfer RNA genes	*trnA-*UGC^a,^^b^	*trnC*-GCA	*trnD*-GUC	*trnE*-UUC	*trnF*-GAA
		*trnfM*-CAU^a^	*trnG*-UCC	*trnH*-GUG^a^	*trnI*-CAU^a^	*trnI*-GAU^a,^^b^
		trnK-UUU^b^	*trnL*-CAA^a^	*trnL*-UAA^b^	*trnL*-UAG	*trnM*-CAU^a^
		*trnN*-GUU^a^	*trnP*-UGG	*trnQ*-UUG	*trnR*-ACG^a^	*trnR*-UCU
		*trnS*-GCU	*trnS*-GGA	*trnS*-UGA	*trnT*-GGU	*trnT*-UGU
		*trnV*-GAC^a^	*trnV*-UAC^b^	*trnW*-CCA	*trnY*-GUA	
	Small subunit of ribosome	*rps2*	*rps3*	*rps4*	*rps7*^a^	*rps8*
		*rps11*	*rps12*^a,^^c^	*rps14*	*rps15*^a^	*rps16*^b^
		*rps18*	*rps19*^a^			
	Large subunit of ribosome	*rpl2*^a,^^b^	*rpl14*	*rpl16*^b^	*rpl20*	*rpl22*
		*rpl23*^a^	*rpl32*	*rpl33*	*rpl36*	
	DNA-dependent RNA polymerase	*rpoA*	*rpoB*	*rpoC1*	*rpoC2*	
Genes for photosynthesis	Subunits of NADH-dehydrogenase	*ndhA*^b^	*ndhB*^a,^^b^	*ndhC*	*ndhD*	*ndhE*
		*ndhF*	*ndhG*	*ndhH*	*ndhI*	*ndhJ*
		*ndhK*				
	Subunits of photosystem I	*psaA*	*psaB*	*psaC*	*psaI*	*psaJ*
		*ycf3*^c^	*ycf4*			
	Subunits of photosystem II	*psbA*	*psbB*	*psbC*	*psbD*	*psbE*
		*psbF*	*psbH*	*psbI*	*psbJ*	*psbK*
		*psbL*	*psbM*	*psbN*	*psbT*	*psbZ*
	Subnuits of cytochrome b/f complex	*petA*	*petB*^b^	*petD*^b^	*petG*	*petL*
		*petN*				
	Subunits of ATP synthase	*atpA*	*atpB*	*atpE*	*atpF*^b^	*atpH*
		*atpI*				
	Subunits of rubisco	*rbcL*				
Other genes	Maturase	*matK*				
	Protease	*clpP*				
	Envelope membrane protein	*cemA*				
	Translation initiation factor 1	*infA*				
	C-type cytochrome synthesis gene	*ccsA*				
Genes of unknown function	Conserved open reading frames	*ycf15*^a^	*ycf68*^a^			

^a^Two gene copies in IRs; ^b^Gene containing a single intron; ^c^Gene containing two introns.

### 3.3. Microsatellite polymorphisms and repeat sequences

**Figure 3.**
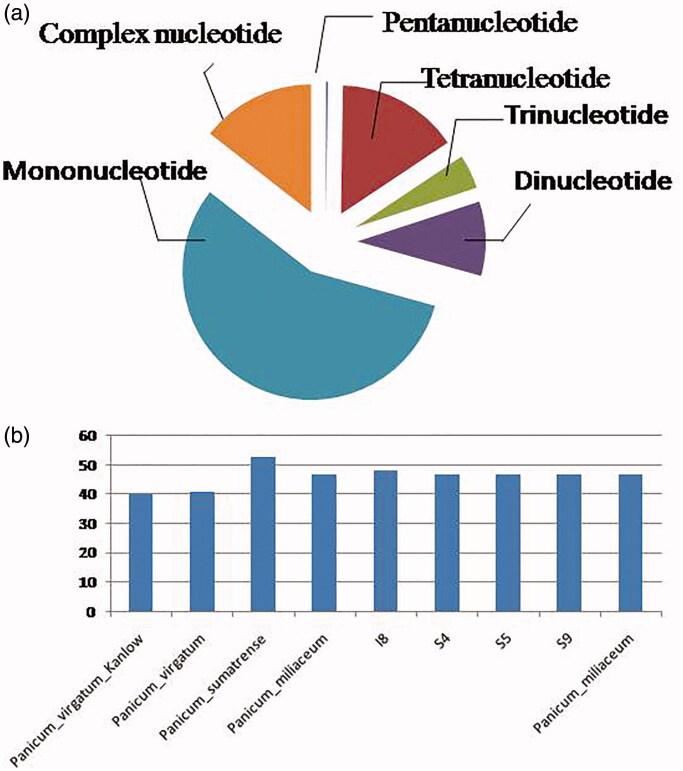
Characterization of simple sequence repeats (SSRs) in the five Common millet (*P. miliaceum*) and samples in this study. (a) Distribution of different SSR repeat motif types, (b) number of SSRs in the whole chloroplast genomes.

Each common millet complete chloroplast genome contained 41 to 53 SSRs with a length of more than 10 bp. Mono-, di-, trin-, tetra-, penta-, and complex nucleotide SSRs were detected in every species, among these SSRs most were l mononucleotide repeats (56.25% of the total occurrences), 60 complex nucleotide (14.4%), 39 Dinucleotide (9.4%), 18 Trinucleotide (4.3%, 64 Tetranucleotide (15.4%), and only one Pentanucleotide (Figure 3 and Table S1). *Panicum sumatrense* included from 6 to 13 more SSR loci in their Whole chloroplast genomes than the other seven samples. the mononucleotide, complex nucleotide, and dinucleotide SSRs averaged 64.8, 10.4, and 5.6%, of all SSRs, respectively. SSRs in common millet Whole chloroplast genomes are especially rich in AT. Nearly all SSRs (100.0%) were mononucleotide A/T repeats, while none C/G mononucleotide SSRs per genome were present. Among dinucleotide SSRs, AT/TA repeats were the most common, trinucleotide SSRs (AGA/TTC) repeats were present in a large number of loci (two, depending on the individual), and depending on species, from 5 to 7 loci contained complex nucleotide repeats (Table S1). AATC, GTAG, AGAA, AGCG, ATTG, AATA, TCGT, and TCCT SSRs were found in all samples, and ATATA was only found in *P. virgatum* in this study (Table S1). The repeat sequence analysis of *Panicum* whole chloroplast genomes found numerous forward repeats, palindromic repeats, and reverse repeats, all of at least 30 bp with a sequence identity ≥90% (Table S2). The repeats ranged from 10 to 238 bp in length and were repeated.

### 3.4. Phylogenetic analysis

**Figure 4.**
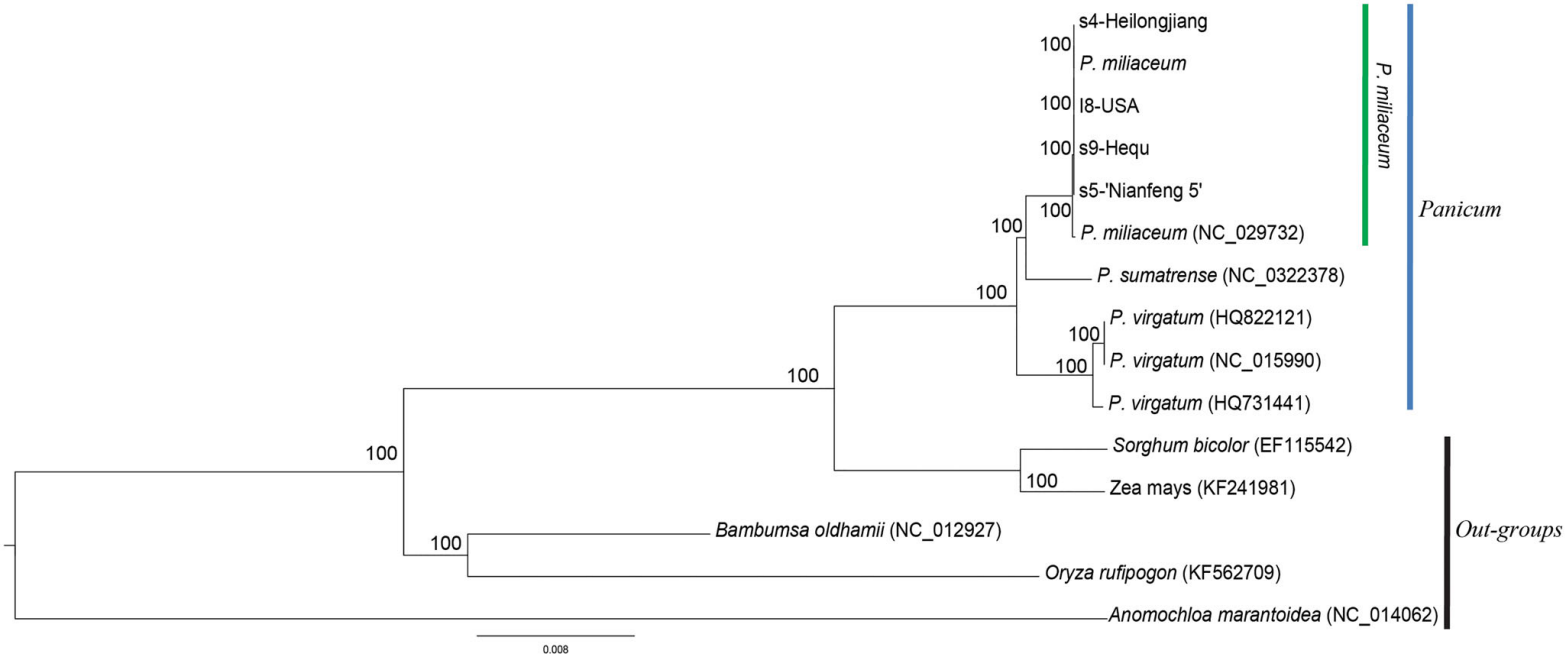
Phylogenetic tree construction of five *P. miliaceum* plus 9 taxa using by maximum-likelihood (ML) based whole cp genome sequences.

We used complete chloroplast genome sequences to analyze the phylogenetic relationships among members of five common millet and closely related species, using as outgroups *Anomochloa marantoidea*, *Oryza rufipogon*, *Bambumsa oldhamii*, *Z. mays*, and *Sorghum bicolor*. The reconstructed phylogeny of all samples divided into seven clades (Figure 4), with members of the *P. sumatrense* joined to the six common millets and distinct from the *P. sumatrense*, irrespective of the dataset. Within *Panicum*, the three species were divided into three clades with 100% bootstrap (BS) support. In common millet, five individuals belong to the same group *J. regia* were closely related with the species *P. sumatrense* with a 100% BS, while three samples of with the species *P. sumatrense* were closed related with 100% BS value (Figure 4). The newly characterized five common millet complete chloroplast genome will provide essential data for further study on the phylogeny and evolution of the genus *Panicum* and of the Gramineae, for molecular breeding, and potential for genetic engineering.

## Data Availability

The data that support the findings of this study are available in NCBI(National Center for Biotechnology Information) at https://www.ncbi.nlm.nih.gov/search/all/?term=NC_029732. The accession number is NC_029732. The raw data have been deposited as a BioProject under accession no. PRJNA658058.
